# Similarities and Differences between EU Platforms in the AHA and AAL Domains from a Software Viewpoint

**DOI:** 10.3390/healthcare10020401

**Published:** 2022-02-21

**Authors:** Matjaž Gams, Žiga Kolar, Zdenko Vuk, Christina Samuelsson, Bernhard Jäger, Erik Dovgan

**Affiliations:** 1 Department of Intelligent Systems, Jožef Stefan Institute, Jamova Cesta 39, 1000 Ljubljana, Slovenia; matjaz.gams@ijs.si (M.G.); ziga.kolar@ijs.si (Ž.K.); zdenko.vuk@ijs.si (Z.V.); 2 Department of Biomedical and Clinical Sciences, Division of Sensory Organs and Communication, Linköping University, SE-581 83 Linköping, Sweden; christina.samuelsson@liu.se; 3 SYNYO GmbH, Otto-Bauer-Gasse 5/14, 1060 Wien, Austria; bernhard.jaeger@synyo.com

**Keywords:** platforms, clustering, artificial intelligence, health, older people, PlatformUptake

## Abstract

The EU PlatformUptake project’s main goal is to investigate the usage of EU open and partly-open platforms in active and healthy aging (AHA) and ambient-assisted living (AAL) domains, from a software viewpoint. The aim of the project was to provide tools for a deeper interpretation and examination of the platforms, gather user feedback, and use it to improve the state-of-the-art approach in the AHA and AAL domains, and define instructions to enhance the platforms within the recommended order. The emphasis is on the software viewpoint for decision makers. In this paper, we present (i) the PlatformUptake methodology for AHA open platform assessments and its main objectives; (ii) clustering of the analyzed platforms; and (iii) the taxonomies generated from the text descriptions of the chosen platforms. With the use of the clustering tools, we present which platforms could be grouped together due to their similarities. Different numbers of clusters were obtained with two clustering approaches, resulting in the most informative two and four cluster groups. The platforms could be rather neatly presented in this way and, thus, potentially guide future platform structuring. Moreover, taxonomies, i.e., decision trees of platforms, were generated to easily determine each specific platform or to find platforms with the desired properties. Altogether, the computer comprehension of the platforms may be important additions to the human way of dealing with the AHA platforms, influencing future design, publications, related work, and research.

## 1. Introduction

Aging “presents itself” as one the greatest trials of the twenty-first century for mankind, in particular in the EU. One study shows that more than 20% of Europeans will reach 65 years of age or more by 2025 [1]. To solve the aging issue, the EU decided to spend additional resources to preserve and reinforce the area of active and healthy aging. In recent years, a substantial number of open source platforms have been developed in active and healthy aging (AHA) and ambient-assisted living (AAL) domains (henceforth referred to as the “AHA domain”). Such solutions present a matter of importance, interest, and investigation of the EU project, PlatformUptake [2]. These platforms, in the context of our project, facilitate the development of digital technology for older people and, thus, comply with software principles, such as interoperability and modularity. They are also successfully applied (e.g., FIWARE, MiBida) in other verticals, such as health care and smart cities. MiBida resulted from an EU-funded project and is currently used by hospitals in the Netherlands (as an EMR type of platform).

However, in the AHA and AAL domains for elder people, up until now, there was no structure or similarity study between the existing platforms. Such organization of the field provides an organized overview of the field, enabling new comprehension, better designs, and better decisions for IT designers, decision makers, and users and physicians, because they easier choose the proper types of platforms.

The PlatformUptake methodology for AHA and AAL open platform evaluation provides tools for deeper interpretation and examination of the platforms, gathers user feedback and uses it to improve the state-of-the-art approach, and defines instructions to enhance the platforms within the recommended order. Over the course of the project, an open information hub was established, providing information and specification on all existing platforms. With the task of raising more awareness on the AHA domain and building more trust between all stakeholders, a massive open online course (MOOC) was created and multiple events were hosted where the stakeholders had the upper hand in decision making [2].

As part of the project, a set of chosen platforms was carefully analyzed by human experts and described in the texts. This paper presents a computer-aided analysis of these platform descriptions. In particular, the computer methods with the use of artificial intelligence provide computer-generated relations among the platforms, structure, and taxonomy. In this paper, the interesting observations provided clues to how many and which clusters, i.e., groups of similar platforms could be consistently found and indicated that there was a rather consistent structure from the software viewpoint. Finally, decision trees and taxonomies, generated with artificial intelligence methods [3], helped distinguish one platform from the other.

The remainder of the paper is as follows: the related work is presented in Section 2. Section 3 presents the main objectives of the project, project methodology, and platforms for older people. Clustering and taxonomies are presented in Section 5 and Section 6. Finally, the paper is concluded in Section 7 with a short recap and ideas for improvement.

## 2. Related Work

Several platforms specialize in various research and application topics. Their analyses and categorizations are of key importance for their end-users as well as for developers of new or existing platforms. Pal et al. [4] analyzed the platforms for the development of agent systems. They analyzed various aspects, such as commercial or open-source availability, and general or special-purpose ones. Figueroa et al. [5] compared platforms for interactive virtual reality applications. They measured a set of parameters (e.g., distance error during object movement) and statistically assessed the results of the compared platforms. A comparison of IoT platforms was presented by Guth et al. [6]. It mainly focused on IoT-specific issues, such as how to present the physical IoT devices in various platforms. Yu and Kim [7] focused on the security of the IoT platforms by analyzing the supported security mechanisms for authentication, data transport, etc. Ali and Abdullah [8] presented an analysis of platforms for online processing of big data. They listed the key features required for processing big data, such as streaming learning type and appropriated machine-learning tools. Several taxonomies were developed, based on platform characteristics—typically based on expert knowledge. For example, Hofer and Karagiannis [9] presented a taxonomy for cloud computing platforms. Blaschke et al. [10] developed a taxonomy of digital platforms.

Although a similar approach can be applied for the assessment of platforms from various domains, it is preferable to use domain-specific platform comparisons. When comparing platforms for older people, Palumbo [11] considered abstraction level, programming model, control type, interaction type, context management, and service management. Baquero et al. [12] analyzed ambient intelligence frameworks in terms of their purposes, architecture, used platform, inclusion of component supervision module, and repository sensor handling. Madureira et al. [13] analyzed middleware platforms for active and healthy aging by specifying a set of features that such platforms should have. Marcos-Pablos and García-Peñalvo [14] analyzed care and assistance ecosystems by considering the groups of ecosystems (health, monitoring, social interaction, reminder, rehabilitation), system actors (older people, disabled, patients, relatives, regulatory parties, research, etc.), allowed modifications (add devices, add services, add software, modify any of them), maturity (conceptualization, pilots, deployed), sensor types (if any), health-related standards (if any), etc. Various taxonomies were also created for platforms for older people. For example, Chiarini et al. [15] developed a taxonomy for mHealth platforms, while Beevi et al. [16] and Byrne et al. [17] presented taxonomies of assisted living systems.

PlatformUptake differs from the modern platform analysis because it focuses on technical, contextual, and business questionnaires, aimed at developers and executives of AHA platforms. The questionnaire answers are used to determine critical success factors for the successful uptake of AHA platforms. The main methodology of the PlatformUptake analysis is described in detail in [18]. In this paper, we upgraded the main project methodology by introducing the machine-learning-based data analysis. More precisely, we applied k-means clustering, hierarchical clustering, principal component analysis, and decision trees to automatically determine and semantically describe meaningful platform groups, as well as to create platform taxonomy.

## 3. Methodology for AHA Platform Assessment

The focus of this study is on the software properties of EU AHA and AAL platforms.

### 3.1. Main Objectives

The main objectives of the PlatformUptake methodology for the AHA platform assessment are [2]:

- To evaluate the progress of implementation, distribution, and advancement of open platforms in the AHA domain with the help of a methodology.
- To establish tools for platform supporters and users for self-evaluation purposes with which they can measure how satisfied and successful they feel with platform usage.
- To interpret the existing platforms by determining how familiar projects influence their progress, viability, and privileges.
- To connect different members of associations and related partners with the same vision—to share common ideas, expertise, and awareness about open platforms.
- To gather feedback from the end-users and to measure the the development and enlargement of the current state-of-the-art models and specifications.
- To educate the end-users as much as possible in order to maximize the their experiences in using the existing platforms.

### 3.2. Methodology

The PlatformUptake methodology for AHA platform assessment strives to study the usage of open and semi-open platforms in the active and healthy domain and it examines the relationship between these platforms. The methodology follows strict procedures to identify the key ingredients for platform growth and expansion across Europe. It achieves this by considering the feedback of the end-users and by observing the ecological environment and stakeholder communities [2].

To study the use of open platforms for the considered methodology, data have to be collected and processed from various European projects that use such platforms at the core of the fundamentals. After the data are acquired, the methodology proposes evaluation instructions for state-of-the-art models from numerous inter-connected platforms. It considers specialized, institutional, commercial, and juridical facets [2].

It also provides utility tools for all associated partners in order to share the best state-of-the-art methods, innovative solutions, and ideas to enhance the existing platforms. The first useful utility developed during the time of the project was a new tool that allowed platform developers and end-users to self-evaluate their experiences. The second utility was the open information hub that allowed the user to view all information about the platforms in one place. Finally, the establishment of MOOC allowed the stakeholders and other interested parties to gather and share more knowledge about the open platforms [2].

### 3.3. Platforms

Within the development of the methodology, 18 platforms for older people were analyzed. The main purpose was to allow the interoperability of connected systems, such as devices, services, and applications. The majority of them were developed with focus on the AAL and AHA fields, while some (EkoSmart, FIWARE, INTER-IoT, sensiNact, SOFIA2) are more general-purpose; thus, they also support other fields. These platforms were selected from a larger set of identified relevant platforms according to the knowledge of the members of the EU PlatformUptake project. Although the countries of their development were not taken into account during the platform selection and analyses, the European lineage was needed for all of them due to the focus of the EU project.

1. ACTIVAGE consists of a set of techniques, tools, and methodologies for interoperability between heterogeneous IoT platforms and an open framework for providing semantic interoperability of IoT platforms for AHA, while addressing trustworthiness, privacy, data protection, and security [19]. The platform was developed in Italy, Spain, France, UK, Germany, Finland, and Greece.
2. The AMIGO project develops open, standardized, interoperable middleware and attractive user services for the networked home environment [20]. The platform was developed in Switzerland and Italy.
3. The aim of the AmIVITAL project is to support the active and healthy aging (AHA) domain with new ICT technologies and ambient intelligence gadgets. Such devices allow better control for those with chronic disabilities, promote healthy lifestyles, and provide better support for autonomous living [21]. The platform was developed in Spain.
4. BeyondSilos aims at further spreading ICT-enabled, joined-up health and social care for older people, by developing, piloting, and evaluating integrated services based on two generic pathways in a multicentric approach, making extensive use of knowledge and experience gained among early adopters of integrated E-care in Europe [22]. The platform was developed in Switzerland and Italy.
5. The purpose of the EkoSmart program is to develop a smart city ecosystem with all of the support mechanisms necessary for efficient, optimized, and gradual integration of individual areas into a unified and coherent system of value chains, where care for older people is one of the core modules of the smart city, encapsulating every aspect of older people [23,24]. The platform was developed in Slovenia by 25 partners from academia, the medical field, and the industry.
6. The FIWARE foundation is the legal independent body providing shared resources to help achieve the FIWARE mission by promoting, augmenting, protecting, and validating the FIWARE technologies, as well as the activities of the FIWARE community, empowering its members, including end-users, developers, and the rest of the stakeholders in the entire ecosystem [25]. The platform was developed in Belgium, Brazil, Switzerland, Germany, Spain, Finland, France, Hungary, Italy, Netherlands, Portugal, Sweden, United Kingdom, and Colombia.
7. GIRAFF+ is a complex system that can monitor activities in the home using a network of sensors, both in and around the home, as well as on the user’s body [26]. The platform was developed in Sweden, Spain, Italy, Portugal, UK, and Slovenia.
8. INLIFE aims to prolong and support independent living for older people with cognitive impairments, through interoperable, open, personalized and seamless ICT services that support home activities, communication, health maintenance, travel, mobility and socialization, with novel, scalable and viable business models, based on feedback from large-scale, multi-country pilots. An older person wears a sensor and the integrated system provides him/her basic care functions [27,28]. The platform was developed in United Kingdom, Slovenia, Ireland, Austria, Netherlands, Spain, Sweden, and Greece.
9. In the absence of global IoT standards, the INTER-IoT results allow any company to design and develop new IoT devices or services, leveraging the existing ecosystem, and bringing them to market quickly [29]. The platform was developed in Spain, Italy, Netherlands, UK, Slovenia, Poland, and France.
10. OASIS introduces an innovative, ontology-driven, open reference architecture and platform, which enables and facilitates interoperability, seamless connectivity, and sharing of content between different services and ontologies in all application domains relevant to older people and beyond [30]. The platform was developed in Italy, Austria, Belgium, Bulgaria, Switzerland, China, Greece, Spain, Germany, Netherlands, Mexico, Romania, and United Kingdom.
11. PERSONA aims at advancing the paradigm of ambient intelligence through the harmonization of ambient-assisted living (AAL) technologies and concepts for the development of sustainable and affordable solutions for the social inclusion and independent living of senior citizens, integrated in a common semantic framework [31]. The platform was developed in Switzerland and Italy.
12. REACH2020 represents a solution that seeks to prevent older people from loss of function and a decline from being able to perform daily living activities, independently, leading ultimately to entering long-term care [32]. The platform was developed in Germany, Netherlands, Switzerland, Denmark, Poland, and Sweden.
13. sensiNact is a horizontal platform dedicated to IoT and is particularly used in various smart city and smart home applications. sensiNact aims to manage IoT protocol and device heterogeneity, and provides synchronous (on demand) and asynchronous (periodic or event based) access to data/actions of IoT devices, as well as access to historic data with a generic and easy-to-use API [33]. The platform was developed in Austria, Spain, Norway, Croatia, Cyprus, and Poland.
14. SOFIA2 is an IoT enabled middleware platform that allows the interoperability of connected systems. It is multi-language and multi-protocol, enabling the interconnection of multiple devices. It provides publishing and subscription mechanisms, facilitating the orchestration of sensors and actuators in order to monitor and act on the environment [34]. The platform was developed in Italy, United Kingdom, Greece, Germany, Sweden, and Spain.
15. SOPRANO designs and develops highly innovative, context-aware smart services with natural and comfortable interfaces for older people at affordable costs, meeting the requirements of users, family, and care providers, and significantly extends the time one can live independently in his/her home when older [35]. The platform was developed in Switzerland and Italy.
16. UNCAP (Ubiquitous interoperable care for aging people) makes use of solutions and technologies developed in previous research projects to develop an open, scalable, and privacy-savvy ICT infrastructure designed to help aging people live independently, while maintaining and improving their lifestyles [36]. The platform was developed in Austria, Spain, Norway, Croatia, Cyprus, and Poland.
17. universAAL enables seamless interoperability of devices, services, and applications for IoT enabled smart environments. The platform provides the framework for communication, connectivity, and compatibility between otherwise disparate products, services, and devices [37]. The platform was developed in Italy, Austria, Denmark, Netherlands, Croatia, Israel, Spain, Germany, Greece, and Poland.
18. The VAALID (The "Accessibility and Usability Validation Framework for AAL Interaction Design Process") project aims to create new tools and methods that facilitate and streamline the process of creation, design, construction, and deployment of technological solutions in the context of AAL, assuring that they are accessible and usable for senior citizens. The main objective of the project is to develop a 3D-immersive simulation platform for computer-aided design and validation of user-interaction subsystems that improves and optimizes the accessibility features of AAL services for social inclusion and independent living [38]. The platform was developed in Spain, Germany, Greece, and Italy.

## 4. Data Preparation

For all 18 platforms studied in the PlatformUptake project and shortly described in Section 3.3, the text descriptions were obtained and examined with the aim of discovering groups/clusters of platforms and platform taxonomy. Although authors of this study were also members of the PlatformUptake project describing the platforms, the texts describing platforms were defined by a substantially larger group and were provided as input to this study. The texts were obtained by thorough literature search, web page examination, and polling of designers and users alike. The obtained texts were transformed into a computer readable form within the presented study, but nothing was added or modified to preserve the objectivity of the study. After the attributes were extracted from the original texts, the descriptions of the platforms were converted into numeric values of the attributes, e.g., “yes” was converted to 10, “no” to 0, and “partial” to 5. Some features could not be directly converted into numbers, e.g., features with string-type unordered values, e.g., “Any (web)”, “Windows, mobile, Symbian”, “Java”. These features were transformed with one-hot encoding [39] into new features, e.g., “is web”, “is Windows”, “is mobile”, “is Symbian”, “is Java”. After these transformations, 67 features were obtained.

There were also some missing data. The missing values were labeled as “none”, “not sure what you mean exactly”, etc. For each feature, we replaced the missing values with the mean value (see Table 1). For example, if there were ten instances (platforms) and three of them had missing values, the missing value was the sum of genuine values divided by the number of genuine values (7).

The 67 features obtained represent the input data for the clustering algorithms and taxonomies. There was an exception for k-means clustering, where the 67 features were transformed with the principal component analysis (PCA) [40] to get a low number of features, which is more suitable for a visual presentation (e.g., with a 2D graph). PCA transforms a high number of features into a low number of uncorrelated PCA components, where each component is a linear combination of the original features [41].

The analyzed features can be semantically grouped into six categories. The first category is closely related to the IoT (internet of things) devices. It is also related to the healthcare monitoring devices, for example, remote patient monitoring, glucose monitoring, heart-rate monitoring, depression and mood monitoring, etc. Features (1) “Connectivity of heterogeneous IoT devices”, (2) “Remote access to IoT devices”, (3) “Remote control to IoT devices”, (4) “Remote maintenance to IoT devices”, (5) “IoT devices activity log, information and status”, (6) “Onboard analysis, intelligent IoT device”, and (7) “Secure access to IoT devices” belong to the IoT devices category.

The second category can be labeled as a utility category and includes helpful mechanisms for platform functionality. It contains features (8) “Devices lifetime management (software updates, remove bugs, fix security vulnerabilities)”, (9) “Location support if the device’s location is not static”, (10) “Auto-diagnostic features”, and (11) “Implements mechanisms able to make available platform services to third parties outside the Platform”.

The third category is related to interoperability. This category is strategically important because interoperability is one of the key fundamentals for the platforms in the AHA/AAL domains. Features that belong to this category are (12) “Interoperability is implemented using a semantic approach”, (13) “Interoperability is implemented using a syntactic approach”, (14) “Implements interoperability between services and functions defined inside the platform”, (15) “Implements interoperability between devices belonging to the platform”, (16) “Implements SOA web services mechanisms to access interoperability feature”, (17) “Implements Restful web services mechanisms to access interoperability feature”, (18) “Offers facilities to make interoperable new sub-systems, devices, applications, etc.”, (19) “Uses existing and well known common data models to implement interoperability”, (20) ”Security and privacy mechanisms are implemented in interoperability”, and (21) “Results of data analytics can be shared with other data analytics algorithms using the Interoperability feature”.

The fourth category is tied to security, protocols, encryption and permissions. Platforms have to be built on the latest standards that keep them safe and secure. These features are (22) “All the applications only request the minimum set of permissions necessary”, (23) “The applications are registered appropriately in the Platform, and cannot be confused with other apps”, (24) “All inputs from external sources and the user are sanitized and validated. This includes data received via the user interface (UI), inter-process communication (IPC) mechanisms, such as intents, custom URLs, and network sources”, (25) “No sensitive data are shared with third parties unless it is a necessary part of the architecture”, (26) “Data are encrypted on the network”, (27) “All the related web servers ensure maintenance and correction against the main known weaknesses”, (28) “Protocols and cryptographic schemes ensure end-to-end data integrity”, (29) “Communications between Platform to the Internet are secured”, (30) “Only authorized devices can be connected to the Platform”, (31) “Compliance with general data protection regulation (EU) 2016/679 (GDPR) (score from 1 to 5)”, (32) “Data link protocols—SodaPop”, (33) “Data link protocols—NGSI”, (34) “Data link protocols—Data With Open mHealth, HL7 v2&3 FHIR”, (35) “Data link protocols—KNX”, (36) “Data link protocols—web”, (37) “Management protocols”, (38) “Security protocols—https, OAuth2”, (39) “Security protocols—Spring, HTTPS”, (40) “Security protocols—SSL”, (41) “Security protocols—own development”, and (42) “Publish-subscribe patterns and related protocols”.

The fifth category is reserved for data analytics. Data analytics enable the platform providers and developers with a deeper look into the platform functionality and help them improve the platforms. It includes (43) “Implements real-time data analytics”, (44) “Implements predictive data analytics”, (45) “Implements data analytics for anomaly detection”, (46) “Security and privacy mechanisms are implemented for data analytics”, (47) “Some data analytics are specific for the AHA domain”, (48) “Data analytics are included in a Marketplace”, (49) “Data analytics are accessible using REST/SOA API calls”, (50) “Data analytics offer GUI interfaces to display results according to the user group (caregiver, patient, etc.)”, (51) “Implemented data analytics analyze body parameters (heartbeat, blood pressure, etc.)”, (52) “Implemented data analytics analyze environmental parameters (temperature, humidity, luminosity, etc.)”, (53)“Visualization of data”, (54) “Visualization of data”, (55) “Creation of analytics”, (56) “Dashboard available for each user group (caregiver, patient, etc.)”, (57) “Provision of suggestions for better lifestyle and personalized coaching to seniors and their relatives”, and (58) “Provision of suggestions to the caregiver for optimal monitoring and treatment”.

Finally, the remaining category is a system properties category. It contains information about the operating system and type of input. Features (59) “Web application or standalone”, (60) “Input—Standard’, (61) “Input—Touch”, (62) “Input—Other”, (63) “Audio output support”, (64) “Operating systems supported (including mobile)—Java OSGi”, (65) “Operating systems supported (including mobile)—Web”, (66) “Operating systems supported (including mobile)—Desktop”, and (67) “Operating systems supported (including mobile)—Android” belong to this category.

One might consider adding more user-oriented attributes, but for the original PlatformUptake project, the text obtained through several months of intensive source extractions were the ones that served for the input of this study. The attributes were primarily software oriented since most of the platforms are actually meta-platforms, or they at least enable adding practically any user-oriented functionality. These modern platforms are not single-purpose programs, but rather sophisticated flexible software products/frameworks tunable for a particular purpose and use in the AHA and ALL domains.

## 5. AHA Platform Clustering

The 18 analyzed platforms for older people were clustered with two clustering approaches. The goal of clustering is to determine how many and which groups represent the platforms best. The applied clustering algorithms are k-means [42] and hierarchical clustering [43].

### 5.1. K-Means Clustering

The k-means algorithm combines objects into a low number of clusters. Each cluster is represented with the cluster center that is calculated as the mean of the objects that belongs to that cluster. An object belongs to the cluster with the nearest center. As a result, k-means clustering divides the space of objects into Voronoi cells, one for each cluster [44].

The k-means clustering for the platforms should reveal what platforms could be best grouped into clusters. Since there are 67 features, the space is 67-dimensional. The PCA method combines the features into a smaller number of features (most commonly two or three) to ease visual representation. For example, if there are just two features, the instances can be presented with a two-dimensional plane.

Clustering was performed for two to eight clusters in two ways: with and without the standard scaler [45,46]. This scaler removes the mean value and scales the result to unit variance, which is a common preprocessing approach for several machine-learning algorithms [45]. Afterwards, clustering was performed and analyzed. The most informative clusters were obtained when clustering into two and four clusters, therefore these results are presented in Section 5.1.1 and Section 5.1.2.

#### 5.1.1. Platform Clustering into Two Clusters

**Figure 1:**
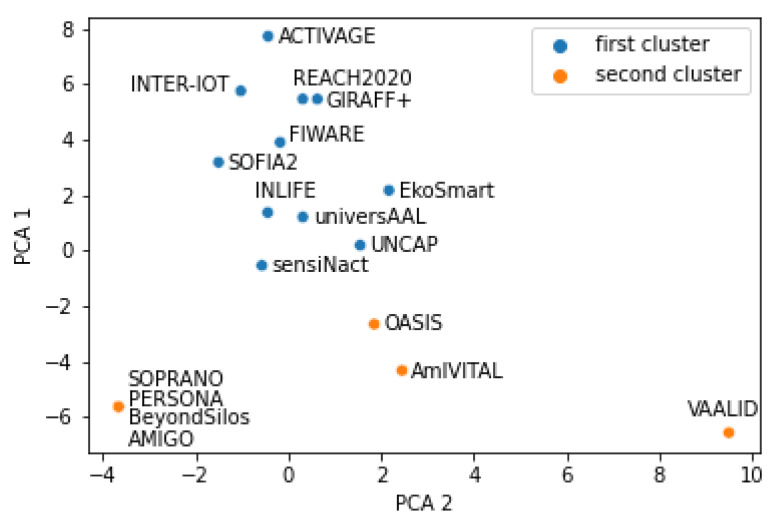
Result of k-means with two clusters and standardized input data.

Figure 1 depicts two clusters generated with the k-means method using the standardized input data. The first cluster is clearly distinguishable from the second since cluster_1 = {x,PCA_1(x)>−2}.

**Figure 2:**
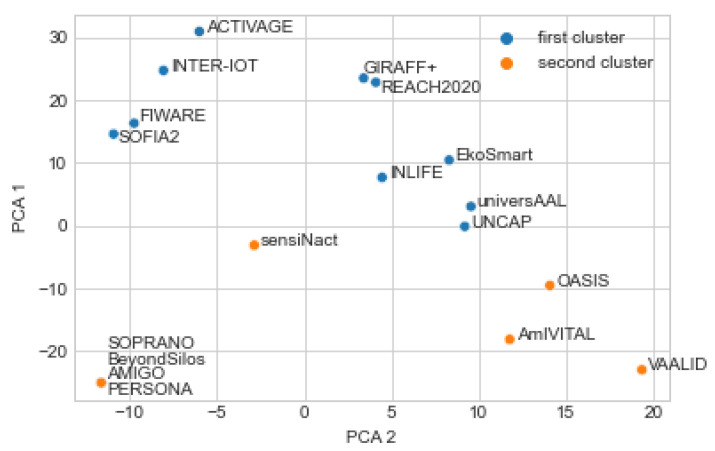
Result of k-means with two clusters and non-standardized input data.

Figure 2 presents another way of clustering into two clusters. This time non-standardized input was used. The obtained clusters are the same as with the standardized input data, with the exception of sensiNact that now belongs to cluster 2. This indicates that there is some disagreement between the two ways of clustering regarding the sensiNact platform, while there is an agreement about all remaining 17 platforms.

It should be noted that there are only 15 dots visible in Figure 1 and related figures although there are 18 platforms. The reason is that four dots are overlapped at the left down corner of the figures and, therefore, are not seen separately. These platforms are: AMIGO, BeyondSilos, PERSONA, SOPRANO. These four platforms are the most similar to each other, as the first clustering results indicate. The reason for similarity is that the values of all 67 features are identical.

The platforms are therefore neatly divided into:

- cluster 1: ACTIVAGE, EkoSmart, FIWARE, GIRAFF+, INLIFE, INTER-IoT, REACH2020, SOFIA2, UNCAP, universAAL
- cluster 2: AmIVITAL, OASIS, VAALID, AMIGO, BeyondSilos, PERSONA, SOPRANO

sensiNact, as mentioned, is in one clustering approach in the first, and in the other clustering approach in the second cluster; therefore, it is not clearly distinguishable by the applied clustering approaches.

#### 5.1.2. Platform Clustering into Four Clusters

**Figure 3:**
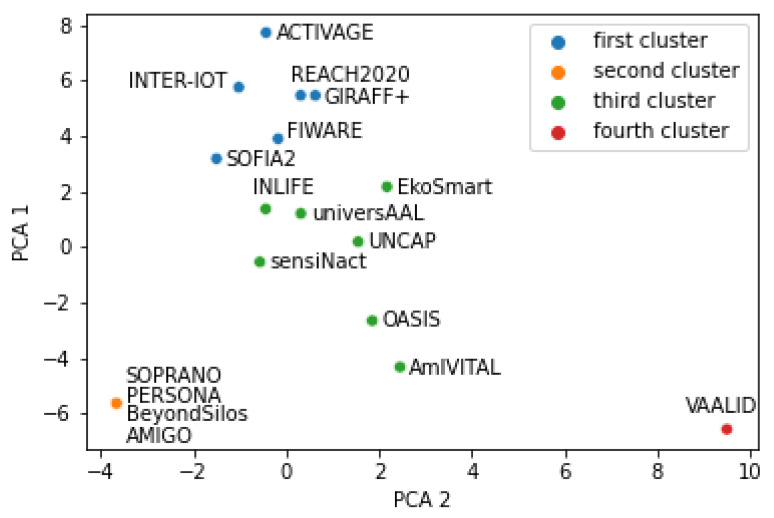
Result of k-means with four clusters and standardized input data.

**Figure 4:**
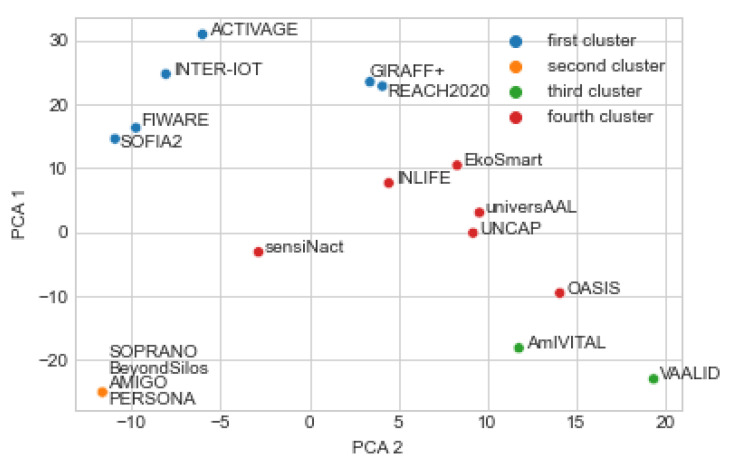
Result of k-means with four clusters and non-standardized input data.

Clustering into four classes is presented in Figure 3 and Figure 4. Similar to two clusters, results in Figure 3 were obtained with standardized input data, while results in Figure 4 were obtained with non-standardized input data. The following four clusters were found:

- ACTIVAGE, FIWARE, GIRAFF+, INTER-IoT, REACH2020, SOFIA2;
- AMIGO, BeyondSilos, PERSONA, SOPRANO;
- VAALID, AmIVITAL;
- EkoSmart, INLIFE, OASIS, sensiNact, UNCAP, universAAL.

By comparing Figure 3 and Figure 4, one difference is observed: AmIVITAL, therefore reasonably consistent grouping is again observed by two different sets of input data.

It is possible to visually inspect the four figures (Figure 1, Figure 2, Figure 3 and Figure 4) in different ways. For example, one question is—what differences were introduced by transitioning from two to four clusters? Another interesting analysis is whether the two main clusters could easily be reconstructed from the four clusters. Positive answers should provide an indication that the presented clusters are quite consistent according to the AI methods.

Figure 3 is generated with the same input data as Figure 1, but this time with the number of clusters set to four. Cluster 1 from the two-cluster generation corresponds to clusters 1 and 3 in the four-cluster generation. There are two exceptions, though: in the four-cluster generation, platforms AmIVITAL and OASIS belong to the third instead of to the second or fourth cluster, as it would in a perfect match.

**Figure A1:**
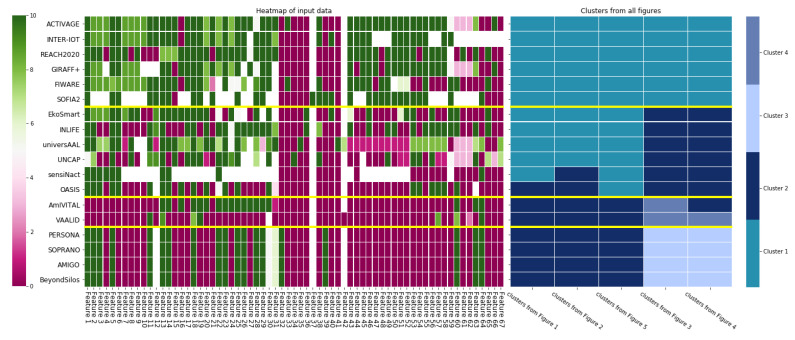
Heat map of input data and heat map of clusters from Figure 1, Figure 2, Figure 3, Figure 4 and Figure 5.

The second observation is that, when comparing Figure 2 and Figure 4, cluster 1 of the two-cluster generation corresponds to clusters 1 and 4 in the four-cluster generation, with the exception of sensiNact and OASIS, which are in the fourth cluster in case of the four-cluster generation, but are in the second cluster in case of the two-cluster generation. Nevertheless, the four clusters reasonably correspond to the two clusters. These observations are also confirmed with Figure A1.

### 5.2. Hierarchical Clustering

Another method for clustering similar objects together is hierarchical clustering. It starts with each object being an independent cluster. Afterwards, it combines the two most similar clusters together and repeats this step until only one cluster remains. The resulting representation of the hierarchically organized clusters is a dendrogram [47], where each cluster is represented by a horizontal line. The line lengths represent the differences between the clusters—the longer the distance, the more different the clusters; the smaller the distance, the more similar clusters.

**Figure 5:**
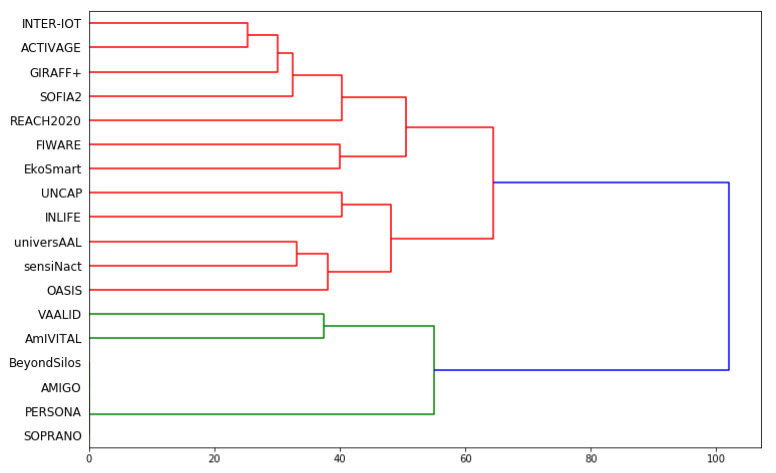
Results of hierarchical clustering of the 18 EU AHA platforms.

Figure 5 shows the dendrogram for the hierarchical clustering. There are two main clusters. One is red-colored and the other is green-colored. The same four platforms are treated as one, since their descriptions are computer indistinguishable: AMIGO, BeyondSilos, PERSONA, SOPRANO. They are at the bottom of Figure 5. They also correspond to the second cluster in Figure 4. Cluster 3 from Figure 4 consists of VAALID and AmIVITAL, and corresponds to the other green-marked platforms in Figure 5.

If four clusters were obtained with hierarchical clustering, they corresponded to the four clusters from Figure 4, with the only exception of EkoSmart, which switched between the first and the fourth cluster, according to the nomenclature of Figure 4.

Another comparison shows that the two clusters obtained in hierarchical clustering were compatible with the k-means clustering with two clusters, as presented by Figure 1, with the exception that OASIS belonged to the other cluster.

Therefore, there is, again, quite a consistent grouping of the 18 platforms with hierarchical clustering (see also Figure A1).

### 5.3. Interpretation of Results

A heat map was utilized to illustrate why an individual platform belongs to a particular cluster. A heat map is a graphical representation of data where values are depicted by colors [48]. The variations of colors may be by hue or intensity, giving a reader a better understanding of how the data varies over space. A heat map of all input data (18 platforms and 67 features) is shown in Figure A1 (left subfigure) and a heat map of clusters from Figure 1, Figure 2, Figure 3, Figure 4 and Figure 5 can be seen in Figure A1 (right subfigure). Input data on the left subfigure contains real numbers in an interval (0, 10); hence, there is a color range that varies between pink (value 0) and green (value 10). Yellow lines on the left subfigure represent the border between two clusters. On the right subfigure, there are five platforms that always correspond to cluster 1, which is a unique property of the right subplot. These platforms are ACTIVAGE, INTER-IoT, REACH2020, GIRAFF+, FIWARE, and SOFIA2. The reason for this can be found on the left heat map. The five platforms distinguish themselves from other platforms because they mostly have green-colored values of features 43–59 (for feature indexes see Section 4). Clusters 3 and 4 have common values in features 33–67, with four exceptions: features 39, 57, 60, and 62. They are correctly classified to the corresponding clusters in 4 out of 5 figures on the right subfigure. Clusters from Figure 5 correspond to clusters 1 and 2 on the right subfigure. Cluster 2 has a different structure of data in features 33–59 than cluster 1 with an exception of features 39, 42, and 57.

In a similar way, we can interpret the importance of each feature to each PCA component. Feature importance is proportional to the absolute value of the feature coefficient [49,50]. Feature contribution over PCA1 and PCA2 can be seen in Table 2 and Table 3 respectively. These tables show that the first principal component (PCA1) has the highest positive associations with feature “Implements Restful web services mechanisms to access interoperability feature”, which belongs to an interoperability category, and several features related to data analytics. The second component (PCA2) has large negative associations with features belonging to categories closely related to the IoT devices, security, protocols, encryption and permissions, and interoperability. In summary, since the PCA1 component is the most important (PCA2 only tries to explain the variance that was not explained by PCA1) and several features on data analytics are associated with PCA1, the use of the PCA method suggests that the data analytics features have the highest influence on the variance of the analyzed data.

In total, seven PCA components were calculated, but only the first two (PCA1 and PCA2) were used for the visualization of clusters. This is because components PCA1 and PCA2 contained the highest explained variance ratios, 0.32 and 0.14, respectively. The remaining components had values ≤0.09. The total explained variance of seven PCA components was equal to 0.8334, which is near the preferable threshold (0.8) to prevent overfitting of the data.

## 6. AHA Platform Taxonomy

### 6.1. Taxonomy

Taxonomy is the practice and science of categorization or classification based on discrete sets. It is a hierarchical classification, in which entities are organized into groups or types. Many taxonomies are often hierarchies in the forms of tree structures. Creating taxonomies often corresponds to training a decision tree from the data, where each leaf in the tree corresponds to a specific object, e.g., a specific plant species, or in our case, a platform [51].

**Figure A2:**
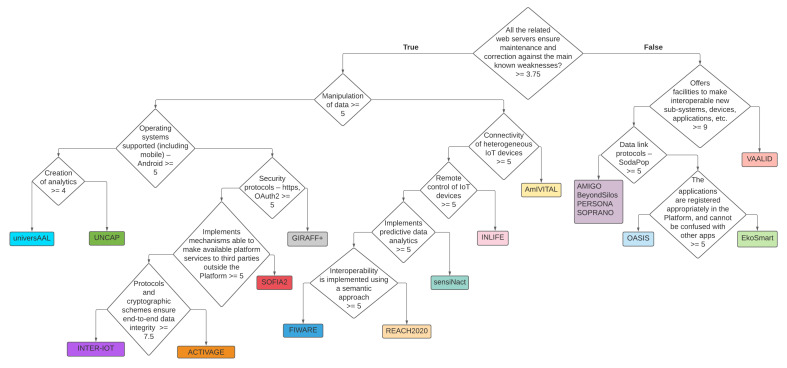
Taxonomy generated from standardized input data.

**Figure A3:**
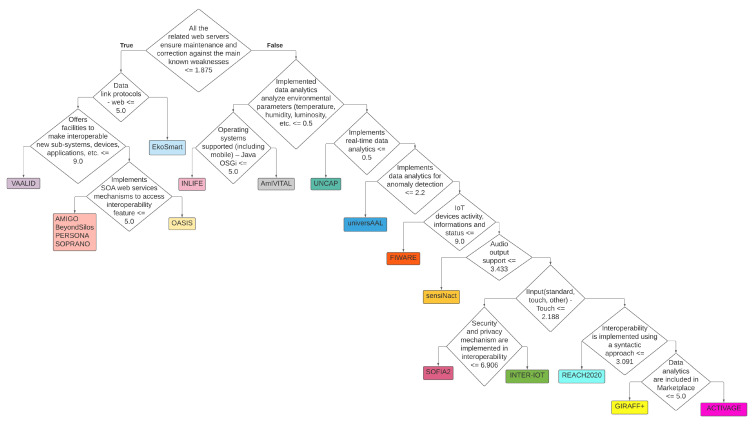
Taxonomy generated from non-standardized input data.

Figure A2 and Figure A3 represent the generated taxonomy on the analyzed platforms, where Figure A2 used standardized input data while Figure A3 used non-standardized input data. Starting from the root in Figure A3 (the top of the decision tree), which contains all the platforms, the platforms split into the left and the right nodes based on the feature/question: “All the related web servers ensure maintenance and corrections against the main known weaknesses ≤ 1.875?”. This procedure of splitting platforms, with the most significant question, repeats, until ideally there is just one platform left, i.e., all other platforms do not correspond to the set of questions, except the one. For example, the yellow leaf corresponds only to the AmIVITAL platform and no other. Its parent node splits platforms looking at the feature named “Operating systems supported (including mobile)—Java OSGi”. If the value is ≤ 5, it continues over its left arrow; if the value is > 5, it continues over its right arrow, in this case for a leaf “AmIVITAL”. Therefore, the features (questions) leading to the yellow node, i.e., the AmIVITAL platform, are:

- All the related web servers ensure... ≤ 1.875;
- Implemented data analytics analyze environmental… ≤ 0.5;
- Operating systems supported (including… ≤ 0.5.

In a similar way, all descriptions of the platforms can be obtained from the generated tree, best differentiating between them. The only exception is the second node from the left, where it is not possible to distinguish between the four platforms. While the previous features lead to all four of them, the algorithm is not able to create further questions to differentiate between them by using additional features.

With the exception of the four platforms, AMIGO, BeyondSilos, PERSONA, SOPRANO, which are so similar in the description language that they are indistinguishable, other platforms are distinguishable, meaning that the chosen way of creating features from the text descriptions discriminates well.

The experiments therefore confirm that it is possible to distinguish the platforms with the exception of the four mentioned. Moreover, the experiments indicate that some features, such as “All the related web servers ensure maintenance and corrections against the main known weaknesses ≤ 1.875?” are highly discriminative since they most commonly appear at the root of the generated tree.

**Figure 6:**
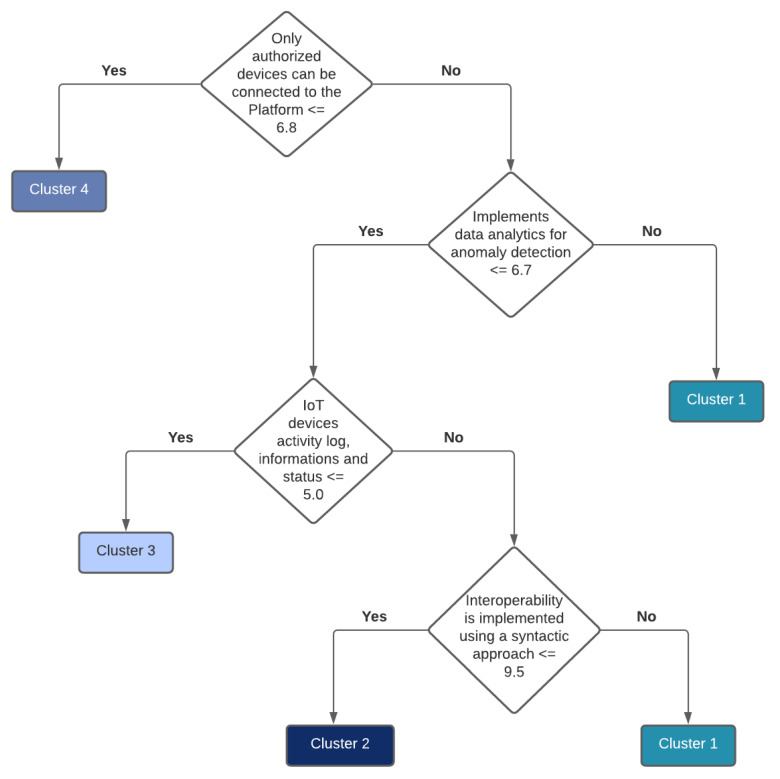
Taxonomy for four clusters from Figure A1.

**Figure 7:**
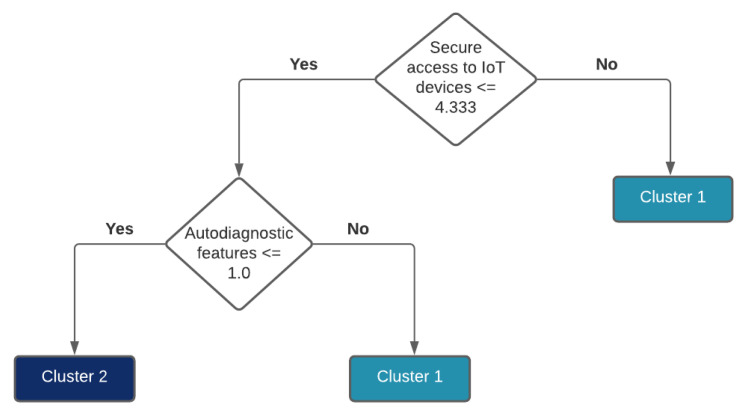
Taxonomy for two clusters from Figure 5 (in line with clusters from Figure A1).

Further experiments indicate that a taxonomy can be used to distinguish different clusters. To accomplish this, the platform names have to be replaced with a corresponding cluster number and used as input to the decision tree. For example, if there are four clusters, every platform is labeled in a range (1, 4). The motivation for this approach is to show which properties are common to the platforms belonging to the same cluster and how clusters differentiate between each other. Figure 6 shows a decision tree for four clusters from Figure A1, while Figure 7 shows a taxonomy for two clusters from Figure 5 (that are in line with clusters from Figure A1). One can observe that two features can distinguish between two clusters (see Figure 7), and four features can be used to distinguish between four clusters (see Figure 6).

### 6.2. Practical Use of Taxonomy

With the obtained clusters and taxonomies, it is possible to classify new platforms in the AHA field. The approach presented in this paper is also applicable to other platforms in other domains. One would just have to create descriptions of the platforms in a similar way and then design taxonomy trees. The algorithm and a corresponding example are as follows:

1. Choose a platform to be classified and describe the platform with features as presented in this paper.
  *Example:* the Insieme platform is developed within the Italian–Slovenian Interreg project ISE-EMH [52], and it is a significantly modified derivative of the medical part of the EkoSmart platform.
2. Classify the chosen platform with taxonomies (see Figure A2 and Figure A3).
  *Example:* the Insieme platform was classified according to taxonomies (Figure A2 and Figure A3) into EkoSmart, which was expected, but also into INLIFE, which was not expected.
3. If both taxonomies classify the chosen platform into the same platform, the result of clustering is the same as presented in Figure 1 and Figure 4.
  *Example:* the classification results from taxonomies (Figure A2 and Figure A3) differ for Insieme; therefore, Step 3 is not relevant for Insieme.
4. If taxonomies (Figure A2 and Figure A3) classify differently, check the clusters in Figure 1, Figure 2, Figure 3, Figure 4 and Figure 5, and observe the obtained results.
  *Example:*
  - In Figure 3, Figure 4 and Figure 5, EkoSmart and INLIFE belong to the same cluster; therefore, Insieme, at least, to a great point, belongs to cluster 1.
  - In Figure 2, EkoSmart and INLIFE belong to clusters 1 and 2; therefore, Insieme belongs to either cluster 1 or 2.
5. Provide statistics on how often the taxonomy classification falls into the same cluster, determining the clustering of the chosen platform.
  *Example:* the Insieme platform belongs to cluster 1 with 4/5; and with 1/5 probability to cluster 2. It is most similar to EkoSmart and Insieme.

The result might come as some surprise because Insieme, EkoSmart, and INLIFE have different functionalities:

- The original EkoSmart name denoted a platform for a smart city where the same-named platform was the EkoSmart part dedicated to older people and people with health issues, regardless of age.
- The INLIFE platform was dedicated to older people and provided basic care support, functionally using data from wearables.
- Insieme is a platform dedicated to people of any age. It provides info on where a user with a medical issue can obtain quick access to institutions, videos, pages, and forums, a kind of expert, a local “doctor Google”.

It seems that our classification algorithm has some mixed results; 4/5 of the decisions of Insieme belong to cluster 1. Since all three platforms have the same SW designer, i.e., the JSI team, a potential explanation is that the properties of the software of the platform play an important role in these classifications.

## 7. Discussion and Conclusions

We systematically described and evaluated 18 EU chosen platforms in the AHA and AAL domains, because they are facilitators of technology that could be used by older people to improve the quality of their daily lives (e.g., to become more independent in their daily lives). The need for this study stems from the early stage of this field, where no systematic categorization or universal support tools exist. While basic AHA and AAL domains have existed for quite some time, new generations of these platforms have provided several new functions that were nonexistent in previous platforms, such as meta-structure, connectivity, and interoperability, which enable integrated use. As such, they provide opportunities to structure, cluster, and organize the platforms, which is the goal of this study.

The above approach is basically independent of the platforms studied, and could be applied to other domains as well. However, it is of essential importance that the descriptions of the platforms are performed in the same systematic way, objectively and independently of the second stage, to eliminate subjectivity. In the PlatformUptake project, the descriptions were designed on their own, as part of the EU project, without keeping in mind that this additional study would be performed. Creating such descriptions is a tedious task on its own since each platform is designed with its specifics, and is also presented typically by the authors on their web pages in their own styles, wording, and renderings, which make the descriptions non-systematic, heterogeneous, and difficult to unify. To cope with this issue, a uniform questionnaire was designed and filled for each platform, while polling the designers and users alike.

Based on the text descriptions of the 18 platforms, transformed into a computer readable form, the platforms were clustered with three clustering procedures, two k-means, and one hierarchical, and were included into two taxonomies for classification purposes. The input was based on the features generated from the text descriptions of the platforms. We conclude that the platforms could be clustered into two and four clusters in the following way:

- There are four platforms with similar text descriptions that the computer-generated features could not distinguish among them: AMIGO, BeyondSilos, PERSONA, SOPRANO.
- The split into two clusters was:
  - cluster 1: ACTIVAGE, EkoSmart, FIWARE, GIRAFF+, INLIFE, INTER-IoT, REACH2020, SOFIA2, UNCAP, universAAL.
  - cluster 2: AmIVITAL, OASIS, VAALID, AMIGO, BeyondSilos, PERSONA, SOPRANO.
- The split into four clusters was as follows:
  - cluster 1: AmIVITAL, EkoSmart, INLIFE, OASIS, sensiNact, UNCAP, universAAL.
  - cluster 2: ACTIVAGE, FIWARE, GIRAFF+, INTER-IoT, REACH2020, SOFIA2.
  - cluster 3: AMIGO, BeyondSilos, PERSONA, SOPRANO.
  - cluster 4: VAALID.

Based on this study, any AHA platform could be put into one of the observed clusters, thus presenting a better understanding of its type.

Clustering into two groups displayed greater consistency than clustering into four groups, which is to be expected. Moreover, clustering determined the platforms close to the centroids of the clusters and the ones at the borders that were sometimes clustered in different clusters based on the clustering method applied.

The taxonomy analysis indicates that, again, the four platforms are indistinguishably similar, and that besides the four platforms, all other platforms are distinguishable as separate entities. Any new AHA platform can be classified according to the two taxonomies presented in order to find the most similar existing platform. If both taxonomies lead to the same platform, this platform enables determining the most similar cluster. If the two taxonomies result in two different platforms, then the conclusion about proper clusters of the observed platform can be derived from the clusters according to the proposed algorithm.

Another derivation from this study is that some features, such as the one appearing at the root of the taxonomies, separate the platforms into two subgroups. The most important feature according to our analysis is: “All the related web servers ensure maintenance and corrections against the main known weaknesses ≤ 1.875?”. This indicates that some of the platforms provide proper support, i.e., maintenance and corrections, and that the other group of platforms is more of an academic nature.

In summary, the presented clustering approach and taxonomies for platforms for older people enable integration and structuring of the field of EU platforms for older people. In future designs and implementations of the AHA platforms, one could use clustering and taxonomies to systematically compare them to the most similar platforms and groups of platforms.

In future work, a deeper analysis of the clustering is required. First of all, human experts should figure out the human meaning of the clusters. At this point, the computer methods propose clusters from 67 features, but it is possible that, for example, humans would prefer different features for taxonomies, which is more human comprehensible. Second, more classifications of new platforms should be provided and some statistics with it. Third, instead of primarily relying on software attributes of platforms, semantic and functional attributes could be applied, e.g., the practical user-oriented functions that the platforms provide. In this case, the platforms would be grouped and ranked due to their actual uses. However, it should also be noted that most of the platforms are actually meta-platforms, enabling inclusion of any functionality of the elderly; therefore, the essential properties of the platforms would be lost. There is an implicit idea to foster a background or a framework for a potential design of a uniform EU AHA platform, whereas the practical design is left for a potential new project.

## Figures and Tables

**Table 1:** An example of transforming the text description of platforms into proper values for further analysis. “Yes” is transformed into 10, “No” to 0, “Partial” to 5, and the missing values to the mean of the existing values in the column, which is 3.75.

Platform	All Related Web Servers Ensure Maintenance and Correction against the Main Known Weaknesses	Value
ACTIVAGE	Yes	10
AMIGO	No	0
AmIVITAL	Yes	10
BeyondSilos	No	0
EkoSmart	No	0
FIWARE	Not sure	3.75
GIRAFF+	Yes	10
INLIFE	Yes	10
INTER-IoT		3.75
OASIS	No	0
PERSONA	No	0
REACH2020	Partial	5
sensiNact		3.75
SOFIA2		3.75
SOPRANO	No	0
UNCAP	Not sure	3.75
universAAL	Not sure	3.75
VAALID	No, not applicable	0

**Table 2:** Contributions of the top 20 features to the PCA1 component, ordered descending by absolute value.

Feature	Contribution
Implements restful web service mechanisms to access…	0.1968
Implements real-time data analytics	0.1922
Security and privacy mechanisms are implemented for…	0.1906
Implements data analytics, analyzes body parameters…	0.1900
Implements data analytics, analyzes environmental parameters…	0.1862
Onboard analysis, intelligent IoT device	0.1849
Devices, lifetime management (software updates, remove bugs …	0.1833
Creation of analytics	0.1824
Implements data analytics for anomaly detection	0.1806
Secure access to IoT devices	0.1769
Data analytics offer GUI interfaces to display results according…	0.1760
Data analytics are accessible using REST/SOA API calls	0.1684
Visualization of data	0.1591
Web application or standalone	0.1583
All inputs from external sources and the user are sanitized…	0.1577
Only authorized devices can be connected to the platform	0.1530
Implements predictive data analytics	0.1519
Some data analytics are specific for the AHA domain	0.1506
Location support if the device’s location is not static	0.1477
Operating systems supported (including mobile)—Java OSGi	−0.1468

**Table 3:** Contributions of the top 20 features to the PCA2 component, ordered descending by absolute value.

Feature	Contribution
All applications only request the minimum sets of permissions…	−0.2633
Offers facilities to make interoperable new sub−systems…	−0.2511
Remote access to IoT devices	−0.2451
No sensitive data are shared with third parties…	−0.2387
Connectivity of heterogeneous IoT devices	−0.2375
Data are encrypted on the network	−0.2375
IoT device activity logs, information, and status	−0.2304
Protocols and cryptographic schemes ensure end−to−end data…	−0.2224
The applications are registered appropriately in the platform…	−0.2220
Communications between the platform to the internet are secured	−0.2207
Security protocols—Spring, HTTPS	−0.2185
Data link protocols—SodaPop	−0.2135
Remote control of IoT devices	−0.2003
Only authorized devices can be connected to the platform	0.1899
Uses existing and well-known common data models…	0.1770
Compliance with general data protection regulations (EU)…	−0.175
Implements interoperability between devices…	−0.1747
Audio output support	−0.1700
Publish−subscribe patterns and related protocols	−0.1671
Interoperability is implemented using a syntactic approach	−0.1379

## Data Availability

Data sharing are not applicable to this article.

## Abbreviations

The following abbreviations are used in this manuscript:

AI	artificial intelligence
ICT	information and communication technologies
AHA	active and healthy aging
IoT	Internet of Things
PCA	principal component analysis
AAL	ambient-assisted living

## References

[1] (2021). The European Demographic Deficit. https://www.europarl.europa.eu/sides/getDoc.do?pubRef=-//EP//TEXT+IM-PRESS+20080414FCS26499+0+DOC+XML+V0//EN.

[2] (2021). Platform Uptake. https://project.platformuptake.eu/.

[3] Gams M., Gu I., Härmä A., Muñoz A., Tam V. (2019). Artificial intelligence and ambient intelligence. J. Ambient. Intell. Smart Environ..

[4] Pal C., Leon F., Paprzycki M., Ganzha M. (2020). A Review of Platforms for the Development of Agent Systems. arXiv.

[5] Figueroa P., Bischof W.F., Boulanger P., James Hoover H. (2005). Efficient comparison of platform alternatives in interactive virtual reality applications. Int. J. -Hum.-Comput. Stud..

[6] Guth J., Breitenbücher U., Falkenthal M., Leymann F., Reinfurt L. Comparison of IoT platform architectures: A field study based on a reference architecture. Proceedings of the 2016 Cloudification of the Internet of Things (CIoT).

[7] Yu J.Y., Kim Y.G. Analysis of IoT Platform Security: A Survey. Proceedings of the 2019 International Conference on Platform Technology and Service (PlatCon).

[8] Ali A.H., Abdullah M.Z. Recent Trends in Distributed Online Stream Processing Platform for Big Data: Survey. Proceedings of the 2018 1st Annual International Conference on Information and Sciences (AiCIS).

[9] Höfer C., Karagiannis G. (2010). Cloud computing services: Taxonomy and comparison. J. Internet Serv. Appl..

[10] Blaschke M., Haki K., Aier S., Winter R. Taxonomy of Digital Platforms: A Platform Architecture Perspective. Proceedings of the 14th International Conference on Wirtschaftsinformatik (WI2019).

[11] Palumbo F. (2016). Ambient Intelligence in Assisted Living Environments. Ph.D. Thesis.

[12] Baquero R., Rodríguez J., Mendoza S., Decouchant D., Papis A.P.M. (2012). FunBlocks. A Modular Framework for AmI System Development. Sensors.

[13] Madureira P., Cardoso N., Sousa F., Moreira W. My-AHA: Middleware Platform to Sustain Active and Healthy Ageing. Proceedings of the 2019 International Conference on Wireless and Mobile Computing, Networking and Communications (WiMob).

[14] Marcos-Pablos S., García-Peñalvo F.J. (2019). Technological Ecosystems in Care and Assistance: A Systematic Literature Review. Sensors.

[15] Chiarini G., Ray P., Akter S., Masella C., Ganz A. (2013). mHealth Technologies for Chronic Diseases and Elders: A Systematic Review. IEEE J. Sel. Areas Commun..

[16] Beevi F.A., Wagner S., Hallerstede S., Pedersen C. Data quality oriented taxonomy of ambient assisted living systems. Proceedings of the IET International Conference on Technologies for Active and Assisted Living (TechAAL).

[17] Byrne C.A., Collier R., O’Hare G.M.P. (2018). A Review and Classification of Assisted Living Systems. Information.

[18] Carboni A., Russo D., Moroni D., Barsocchi P., Nikolov A., Dantas C., Guardado D., Leandro A., van Staalduinen A., Karanastasis E. Success and Hindrance Factors of AHA-Oriented Open Service Platforms. Proceedings of the 13th International Conference on Computational Collective Intelligence.

[19] (2021). ACTIVAGE. http://www.activageproject.eu/activage-project/#About-ACTIVAGE.

[20] Georgantas N., Mokhtar S.B., Bromberg Y., Issarny V., Kalaoja J., Kantarovitch J., Gerodolle A., Mevissen R. The amigo service architecture for the open networked home environment. Proceedings of the 5th Working IEEE/IFIP Conference on Software Architecture (WICSA’05).

[21] (2021). AmIVITAL. http://www.sabien.upv.es/en/project/amivital/.

[22] (2021). BeyondSilos. https://www.beyondsilos.eu/home/.

[23] (2021). EkoSmart. https://dis.ijs.si/ekosmart/?page_id=137&lang=sl.

[24] Kocuvan P., Tavčar A., Grasselli G., Gams M. Virtual Assistant Aggregator for the Project Electronic and Mobile Health. Proceedings of the 22nd International Multiconference Information Society (IS’19).

[25] (2021). FIWARE. https://www.fiware.org/about-us/.

[26] (2021). GIRAFF+. https://www.age-platform.eu/good-practice/giraffplus-project-smart-homes-technology-tested-real-homes.

[27] Kaimakamis E., Karavidopoulou V., Kilintzis V., Stefanopoulos L., Papageorgiou V. (2017). Development/Testing of a Monitoring System Assisting MCI Patients: The European Project INLIFE. Stud. Health Technol. Inform..

[28] Bizjak J., Gradišek A., Stepančič L., Gjoreski H., Gams M. (2017). Intelligent assistant carer for active aging. Eurasip J. Adv. Signal Process..

[29] (2021). INTER-IoT. https://inter-iot.eu/.

[30] (2021). OASIS. https://cordis.europa.eu/project/rcn/85421/en.

[31] Tazari M.R., Furfari F., Ramos J.P.L., Ferro E. (2010). The PERSONA service platform for AAL spaces. Handbook of Ambient Intelligence and Smart Environments.

[32] (2021). REACH2020. https://reach2020.eu/.

[33] (2021). sensiNact. https://wiki.eclipse.org/sensiNact.

[34] (2021). SOFIA2. https://github.com/Sofia2.

[35] Wolf P., Schmidt A., Klein M. SOPRANO-An extensible, open AAL platform for elderly people based on semantical contracts. Proceedings of the 3rd Workshop on Artificial Intelligence Techniques for Ambient Intelligence (AITAmI’08), 18th European Conference on Artificial Intelligence (ECAI 08).

[36] (2021). UNCAP. https://cordis.europa.eu/project/id/643555.

[37] (2021). univerAAL. https://www.universaal.info/.

[38] (2021). VAALID. https://cordis.europa.eu/project/id/224309.

[39] Okada S., Ohzeki M., Taguchi S. (2019). Efficient partition of integer optimization problems with one-hot encoding. Sci. Rep..

[40] Martinez A.M., Kak A.C. (2001). Pca versus lda. IEEE Trans. Pattern Anal. Mach. Intell..

[41] Jolliffe I.T., Cadima J. (2016). Principal component analysis: A review and recent developments. Philos. Trans. R. Soc. A Math. Phys. Eng. Sci..

[42] Likas A., Vlassis N., Verbeek J.J. (2003). The global k-means clustering algorithm. Pattern Recognit..

[43] Murtagh F., Contreras P. (2012). Algorithms for hierarchical clustering: An overview. Wiley Interdiscip. Rev. Data Min. Knowl. Discov..

[44] (2021). K-Means Clustering. https://en.wikipedia.org/wiki/K-means_clustering.

[45] (2021). Sklearn Preprocessing Standard Scaler. https://scikit-learn.org/stable/modules/generated/sklearn.preprocessing.StandardScaler.html.

[46] (2021). How to Use StandardScaler and MinMaxScaler Transforms in Python. https://machinelearningmastery.com/standardscaler-and-minmaxscaler-transforms-in-python/.

[47] (2021). Hierarchical Clustering. https://www.displayr.com/what-is-hierarchical-clustering/.

[48] (2021). Heat Map. https://www.hotjar.com/heatmaps/.

[49] (2021). PCA Importance 1. https://towardsdatascience.com/pca-clearly-explained-how-when-why-to-use-it-and-feature-importance-a-guide-in-python-7c274582c37e.

[50] (2021). PCA Importance 2. https://support.minitab.com/en-us/minitab/18/help-and-how-to/modeling-statistics/multivariate/how-to/principal-components/interpret-the-results/key-results/.

[51] Piltaver R., Lustrek M., Gams M., Martincic-Ipsic S. (2016). What makes classification trees comprehensible?. Expert Syst. Appl..

[52] (2021). ISE-EMH: Italian-Slovene Ecosystem for Electronic and Mobile Health. http://ita-slo.eu/en/ise-emh.

